# The relationship between health IT characteristics and organizational variables among German healthcare workers

**DOI:** 10.1038/s41598-021-96851-1

**Published:** 2021-09-07

**Authors:** Susanne Gaube, Julia Cecil, Simon Wagner, Andreas Schicho

**Affiliations:** 1 Department of Infection Prevention and Infectious Diseases, University Hospital Regensburg, Regensburg, Germany; 2 LMU Center for Leadership and People Management, LMU Munich, Munich, Germany; 3 Department of Psychology, University of Regensburg, Regensburg, Germany; 4 Department of Radiology, University Hospital Regensburg, Regensburg, Germany

**Keywords:** Health care, Health policy, Health services, Occupational health

## Abstract

Health information technologies (HITs) are widely employed in healthcare and are supposed to improve quality of care and patient safety. However, so far, their implementation has shown mixed results, which might be explainable by understudied psychological factors of human–HIT interaction. Therefore, the present study investigates the association between the perception of HIT characteristics and psychological and organizational variables among 445 healthcare workers via a cross-sectional online survey in Germany. The proposed hypotheses were tested using structural equation modeling. The results showed that good HIT usability was associated with lower levels of techno-overload and lower IT-related strain. In turn, experiencing techno-overload and IT-related strain was associated with lower job satisfaction. An effective error management culture at the workplace was linked to higher job satisfaction and a slightly lower frequency of self-reported medical errors. About 69% of surveyed healthcare workers reported making errors less frequently than their colleagues, suggesting a bias in either the perception or reporting of errors. In conclusion, the study’s findings indicate that ensuring high perceived usability when implementing HITs is crucial to avoiding frustration among healthcare workers and keeping them satisfied. Additionally healthcare facilities should invest in error management programs since error management culture is linked to other important organizational variables.

## Introduction

The adoption of health information technology (HIT), such as electronic health records and clinical decision support systems, has transformed medicine and healthcare and will continue to do so, especially with the rise of new technologies like artificial intelligence (AI). HIT, which can be defined as “any automated or computerized system implemented to aid in the management of health information”^1^, is supposed to reduce costs while improving the quality and effectiveness of healthcare^2,3^. The implementation of HITs can have positive effects on patient outcomes, such as a reduction in adverse events and mortality; however, many studies have found non-significant or mixed results for their benefits^1,4^. At the same time, the introduction of HITs into complex healthcare systems can be disruptive and might even lead to errors that can cause serious harm and death^3,5^. So far, most studies have focused on HITs’ effects on practitioner performance and patient outcomes. However, it is also important to understand the effect HITs have on psychological and organizational variables that might influence users’ performance and patient outcomes in turn.

HITs can be cumbersome and frustrating to use^6,7^ and might result in an adverse condition called *technostress*^8^. Technostress has been defined as the “inability to adapt or cope with new computer technologies in a healthy manner”^9^ and “an IT user’s experience of stress when using technologies”^8^. Technostress has been associated with several adverse organizational outcomes, such as lower productivity, reduced job performance, and increased turnover intention^8^. These negative consequences are influenced by two variables often studied as consequences of technostress: *strain* and *job satisfaction*. Scholars have argued that experiencing technostress can lead to psychological strain^10–12^, which is a person’s response to the exposure to stressors^13^. Examples of techno-stressors are situations in which HITs force the user to work faster and longer or constantly adapt to new technology. However, not every person who is exposed to techno-stressors is affected in the same manner. *Technology self-efficacy*, which is a person’s perceived capability to successfully perform a technologically-related task^14^, has been shown to influence the level of technostress a user experiences^15^. Therefore, technology self-efficacy might moderate the relationship between technostress and IT-related strain. People higher in technology self-efficacy experience lower levels of strain when exposed to techno-stressors and vice versa.

It is well established that higher levels of strain are associated with lower job satisfaction^16–18^. Therefore, healthcare workers’ job satisfaction might also be negatively impacted by the strain caused by exposure to techno-stressors when working with HITs, as has been previously shown in teleworkers^11^. Job satisfaction is the overall evaluative judgment a person has about their job^19^ and is one of the most widely researched organizational variables. Previous studies have also investigated the direct effect of technostress on job satisfaction^8,20–22^. Being exposed to more information than can be handled, having to keep up with rapidly changing and increasingly more complex technology, as well as constant connectivity can leave the user feeling frustrated and dissatisfied. According to one review, the stressor *techno-overload*, which is the technology’s tendency to force a person to work faster and longer, has been especially associated with lower job satisfaction^8^.

The level of technostress experienced by a person can be influenced by individual factors, job-related factors, and technology characteristics. One important technology characteristic associated with technostress, which is also a common problem for health ITs, is poor *usability*^3,5,23,24^. According to the ISO 9241-11:2018, usability is defined as “the extent to which a system, product or service can be used by specified users to achieve specified goals with effectiveness, efficiency and satisfaction in a specified context of use”^25^. Scholars have argued that the three usability features are especially important for technology users: *usefulness*, *ease of use*, and *reliability*^10^. While poor usability, which can create information overload, might cause higher levels of technostress, perceiving the technology as useful, easy to use, and reliable has been associated with lower levels of technostress^10^. Additionally, poor usability is a source of strain^26–28^. However, health ITs with good usability might reduce the user’s perceived strain because they are supposed to make task attainment as easy as possible and therefore decrease workload. Finally, the usability of technology in everyday use might also affect staff job satisfaction^22,29,30^. Users who must interact with poorly designed HITs are likely to be dissatisfied because they might experience workflow interruptions and increased workload. Per its definition, good usability should allow the user to achieve specified goals with satisfaction.

Both work-related strain and job satisfaction are crucial organizational variables. Previous research has identified numerous negative health consequences resulting from persistent work-related strain such as burnout, substance abuse, sleep disturbance, concentration deficits, and many others^31,32^. Strain and these related outcomes can affect workers’ performance^12^ and have been associated with medical errors^18,33,34^. Medical errors are defined as “an act of omission or commission in planning or execution that contributes or could contribute to an unintended result”^35^ and include medication mistakes, wrong diagnoses, and avoidable delays in treatment, among others. Both directions of the relationship between strain and medical errors are plausible: on the one hand, the negative consequences of strain, such as concentration problems, might cause mistakes. On the other hand, realizing that a medical error has occurred might lead to even higher levels of strain. Medical errors are estimated to be among the leading causes of morbidity and mortality among patients^36–38^ and therefore a substantial threat to patient safety. Hence, it is crucial to identify predictors for medical errors, especially factors that have received little attention so far, such as strain caused by the interaction with health IT.

Job satisfaction is another potential factor contributing to medical errors that has not been well-researched. What has been established is the association between job satisfaction and job performance^39,40^. Again, there is evidence for both directions of the relationship: job satisfaction leading to better performance and the other way around. Additionally, low job satisfaction is commonly cited as one of the strongest predictors for counterproductive work behavior^41–43^, which are deliberate and potentially harmful acts towards an organization or its stakeholders such as supervisors, clients, co-workers^44^, and patients. Since both job performance and counterproductive work behavior are related to making mistakes, it might be speculated that there is a direct association between job satisfaction and medical errors.

Naturally, healthcare facilities try to prevent errors altogether to minimize risks and avoid negative consequences. However, realistically, medical errors can never be completely prevented. Error management is an approach aiming to reduce the negative outcomes of errors and increase long-term positive consequences such as learning, innovation, and resilience^45,46^. An organization with a positive error management culture fosters communication about errors, knowledge sharing, and strategies to detect and effectively handle errors^45,46^. In turn, an effective error management culture should lead to better performance^45^ and reduce avoidable errors by applying lessons learned from previous mistakes to improve processes [secondary error prevention^45,46^]. A good error management culture might also have a positive effect on staff members’ job satisfaction because it should reduce their fear of being punished for causing and/or reporting errors^47^.

**Figure 1:**
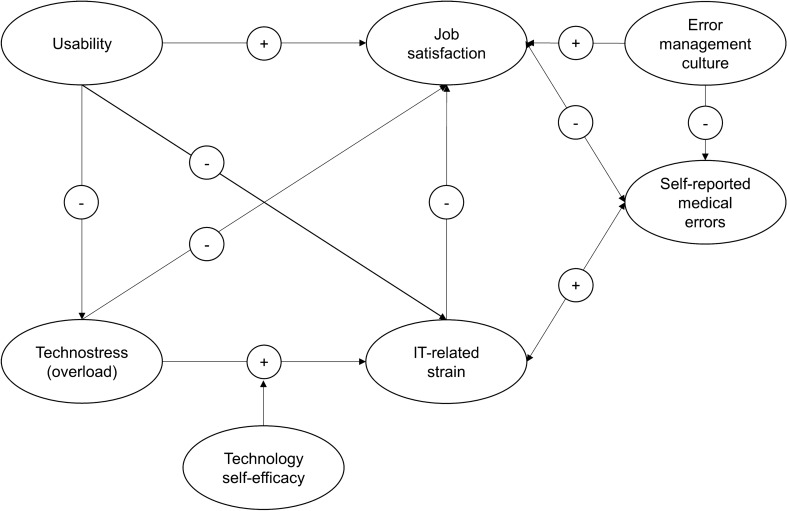
Depiction of the proposed structural equation model. The direction of the assumed association is indicated by + for a positive association or by − for a negative association. The null hypothesis for each assumption is that the association between the variables is zero.

The goal of the present study is to investigate the associations between HIT characteristics (usability and techno-stressors), healthcare workers’ IT-related strain, and job satisfaction. While strain and job satisfaction are important outcome variables themselves, we also want to test if they, combined with healthcare workers’ perceived error management culture, can help to explain self-reported medical errors. These insights might lead to a better understanding of why many studies did not find improvements in patient outcomes after the implementation of HITs. To test the proposed hypotheses (Fig. 1), a nation-wide survey among healthcare workers in German hospitals was conducted.

## Methods

### Participants

The survey for the pre-registered (https://osf.io/r5u43) cross-sectional study was conducted between January and June 2020. Survey invitations were sent via email to 7415 healthcare workers at 43 German hospitals in all 16 states. Most invitations went to the staff at surgical departments, intensive care units, radiology departments, and emergency rooms. A total of 445 participants (response rate 6.00%) completed the online questionnaire on the platform Unipark (Questback GmbH, Cologne, Germany). Participants’ demographics are presented in Table 1.

**Table 1:** Participants demographics.

	% or M (SD)
**Gender (%)**	
Female	44.49
Male	54.83
Non-binary	0.45
NA	0.22
Age (years)	41.90 (11.07)
Work experience (years)	17.63 (11.90)
**Profession**	
Physician	68.99%
Nurse	16.18%
Medical assistant	6.97%
Other	7.87%
**Hospital units**	
Emergency room	4.04%
Intensive care unit	14.61%
Surgical department	39.55%
Functional areas (e.g., radiology)	21.12%
Normal ward	7.87%
Other (e.g., outpatient clinic)	12.81%

*NA* participants preferred not to answer.

*N* = 445.

### Measures

#### Technologies in use

First, participants were asked to indicate which HITs they use most often during their daily work. They could choose multiple options from a preselection and add technologies if needed (see “Supplementary information” for a full list of survey items). Participants were instructed to refer to the selected technologies when answering all other questions. Focusing on a fixed set of frequently used technologies should promote a consistent response to items.

#### Usability

Both the conceptualization and scales to assess the digital HITs’ usability were adapted from a previous study^10^. To measure the three usability features, *usefulness*^48^, *ease of use*^48^, and *reliability*^49,50^, items were rated on a 7-point Likert scale from 1 (*strongly disagree*) to 7 (*strongly agree*). The internal consistency of each subscale (α_usefulness_ = 0.94, α_ease_ = 0.77, α_reliability_ = 0.92) can be considered good to excellent.

#### Technostress

Three technostress creator scales were included in the survey: *techno-overload*, *techno-uncertainty*, and *techno-insecurity*^51^. The two other technostress subscales, *techno-invasion* and *techno-complexity,* were not included. The concept *techno-invasion,* which is the invasive effect of technologies on people’s non-working lives, was considered to be irrelevant for the healthcare context because most healthcare workers do not have access to HITs outside the hospital environment. As *techno-complexity* is very similar to the usability subscale *ease of use*, we decided to only include the usability subscale to keep the survey reasonably short. The included 13 technostress items were answered on a 7-point Likert scale from 1 (*strongly disagree*) to 7 (*strongly agree*). The internal consistency of techno-overload (α_overload_ = 0.85) was good, and of techno-uncertainty (α_uncertainty_ = 0.71) was acceptable. However, the internal consistency of the subscale techno-insecurity (α_insecurity_ = 0.64) was below the acceptable threshold of at least 0.70; therefore, it was removed from further analysis.

#### Technology self-efficacy

Participants’ level of technology self-efficacy was measured with a five-item scale^14^ on a 7-point Likert scale from 1 (*strongly disagree*) to 7 (*strongly agree*). The scale showed acceptable internal consistency (α_self-efficacy_ = 0.76).

#### IT-related strain

Work strain was measured with a scale created by Ayyagari et al.^10^. The response format of the four items was a 7-point Likert scale from 1 (*never*) to 7 (*daily*). The internal consistency of the strain scale was excellent (α_strain_ = 0.92).

#### Job satisfaction

The seven items measuring job satisfaction were adopted from the German version^52^ of the Copenhagen Psychosocial Questionnaire (COPSOQ). The items had a 7-point Likert scale answer format from 1 (*very unsatisfied*) to 7 (*very satisfied*). The scale showed good internal consistency (α_job satisfaction_ = 0.86).

#### Error management culture

We created a short, healthcare-specific, error management culture scale vaguely based on an existing scale^45^. The seven items were answered on a 7-point Likert scale from 1 (*strongly disagree*) to 7 (*strongly agree*). The error management culture scale showed excellent internal consistency (α_error management culture_ = 0.90).

#### Common types of medical errors

Afterwards, respondents selected the three most common medical errors that occur in their work environment from a list of 11 types of potential errors^53^. This list was presented to give participants an idea of what counts as a medical error before asking them about their own errors.

#### Self-reported medical errors

The frequency of medical errors was assessed by asking participants “How often have you made a medical error within the last 3 months?” ranging from 1 (*never*) to 7 (*daily*). In previous surveys, a slightly different version of this item has been used, where participants had to answer with yes or no as to whether they are concerned about having made any medical errors in the last 3 months^34,54^. As this binary measure does not provide any information about the frequency of medical errors, we modified the question and its response scale. The item was followed by a question about the consequences their most recent medical error had for the patient involved, ranging from 1 (*no effect on the patient’s outcome)* to 6 (*patient died*). Healthcare workers also estimated how frequently their colleagues have made medical errors within the last 3 months from 1 (*never*) to 7 (*daily*).

#### Perceived causes for medical errors

Finally, participants were asked to judge the top three causes of medical errors on (a) an individual and organizational level, from a selection of options based on a WHO report on medical errors^55^; and (b) on a technological level, from a list based on usability features^56^.

### Data analysis

Statistical analysis was performed using R version 4.0.4 (The R Foundation for Statistical Computing, Vienna, Austria). To test the proposed hypotheses, structural equation models (SEM) were developed and analyzed with the package lavaan version 0.6-7. The latent variables (i.e., scales such as reliability) are defined in the measurement model by the items they are measured with. The latent variables are analyzed according to the structural model, which tests all the proposed hypotheses. To evaluate the model fit, the following indexes and cutoff levels suggested in the literature^57,58^ were deployed: *χ*^2^/df ≤ 2–3, Root Mean Square Error of Approximation (RMSEA) < 0.06 to 0.08 with confidence intervals, Standardized Root Mean Square Residual (SRMR) ≤ 0.08, Comparative Fit Index (CFI) ≥ 0.95, and Tucker Lewis Index (TLI) ≥ 0.95. The model fit is judged to be good if most cutoff values are met.

### Ethics consideration

The study plan was reviewed and approved by the University Hospital Regensburg Research Ethics Committee (approval number: 19-1611-101). All participants were informed about the purpose of the study and gave informed consent. Participation was completely voluntary, and anonymity was guaranteed. The study complied with all relevant ethical regulations and standards required by the University Hospital Regensburg Research Ethics Committee and the Ethical Principles of Psychologists and Code of Conduct outlined by the American Psychology Association (APA).

## Results

### Descriptive analysis

#### Technologies in use

The three most widely used HITs were hospital information systems, selected by 80.45% of all participants, image archives (80.00%), and electronic health records (71.24%). The complete breakdown of participants’ responses can be found in the “Supplementary information” (Fig. S1).

#### Common types of medical errors

**Figure 2:**
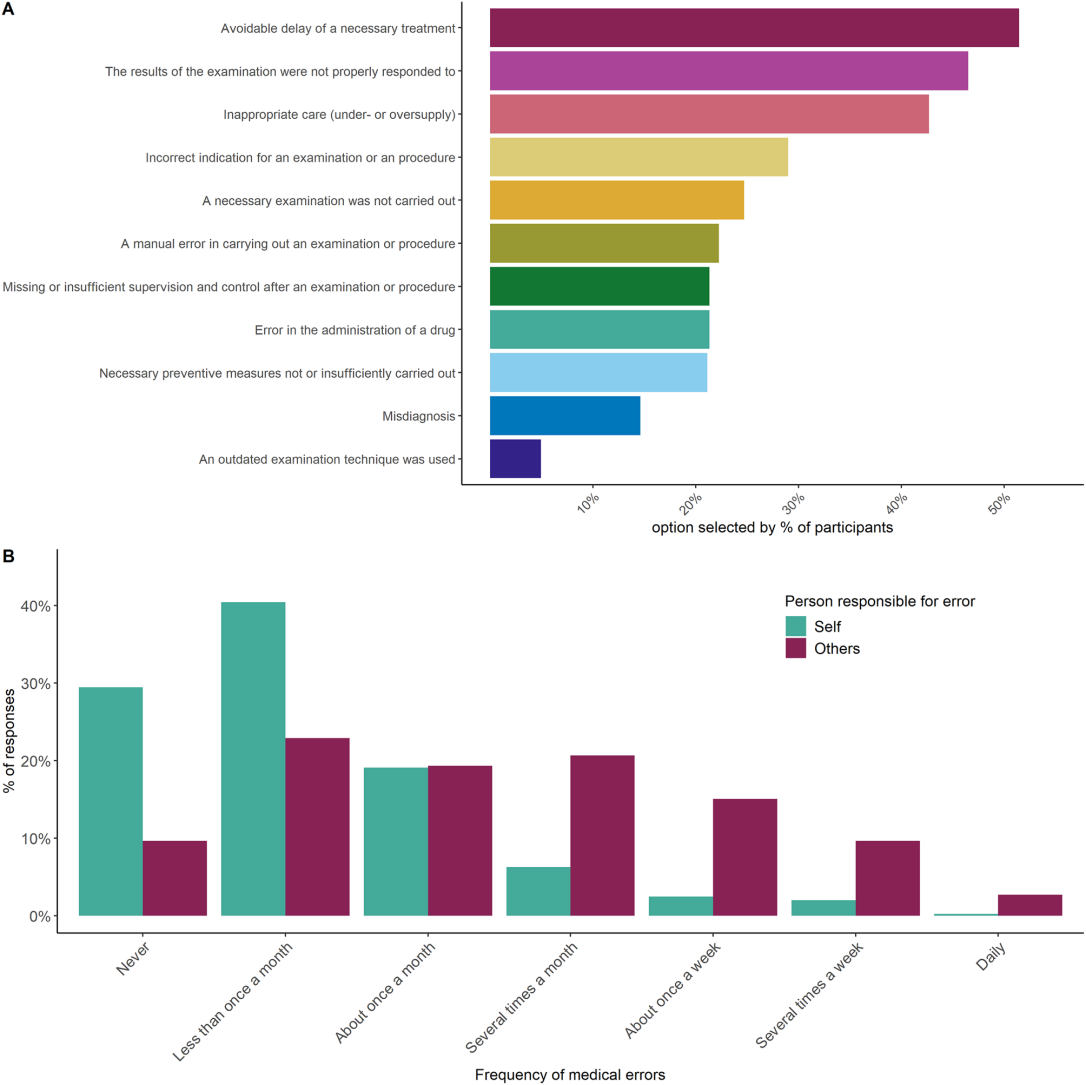
(**A**) Distribution of participants most commonly selected types of medical errors to occur at work. (**B**) Distribution of the self-reported frequency of making a medical error within the last 3 months split into individual’s own errors (self) and errors made by colleagues (others).

In total, 51.46% of the surveyed healthcare workers selected ‘avoidable delay of a necessary treatment’ as one of the three most common medical errors occurring in their work environment, making it the most-reported error. It was followed by ‘the results of the examination were not properly responded to,’ selected by 46.52% of participants, and ‘inappropriate care (under- or oversupply),’ selected by 42.70% of participants. Together, these three accounted for almost half of all selected types of medical errors, making them stand out considerably above any other option. The least-selected types of medical errors were ‘misdiagnosis’ (14.61%), and ‘an outdated examination technique was used’ (4.94%). The full breakdown is reported in Fig. 2A.

#### Self-reported medical errors

**Figure 3:**
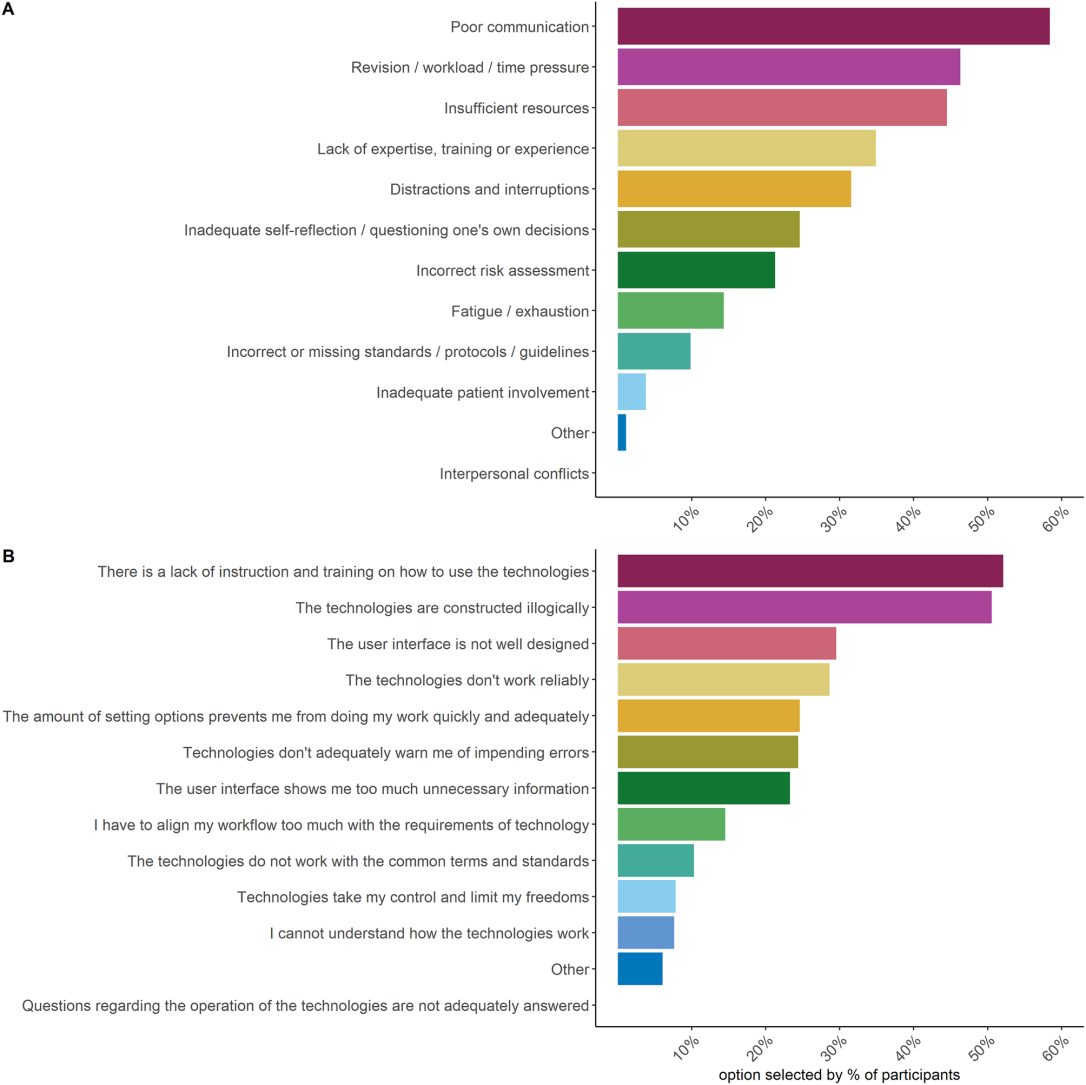
Distribution of participants’ selected top causes of medical errors on (**A**) an individual and organizational level and on (**B**) a technological level.

Figure 2B shows participants’ reported frequency of making medical errors themselves and errors made by colleagues within the last 3 months. Participants reportedly made an error less than once a month on average (*M*_self_ = 2.19, *SD*_*self*_ = 1.14). Most of the participants’ latest medical errors did not harm a patient (49.66%) or only brought minor, temporary harm to a patient (22.47%). Only in 0.45% of the cases was the medical error lethal. Overall, the respondents claimed to make errors less often than their colleagues (*M*_*others*_ = 3.48, *SD*_*others*_ = 1.58, Wilcoxon test: *V* = 2508, *p* < 0.001). There was a strong contrast between the reported frequency of their own errors and the errors of others. A total of 308 (68.90%) participants rated the occurrence of errors made by their colleagues to be higher than their own, 115 (25.73%) regarded the error rate to be the same, and only 22 (4.92%) healthcare workers believed to have made mistakes more often than their colleagues. Figure 3 shows the breakdown of participants’ selected causes for medical errors on both an individual/organizational level (Fig. 3A) and on a technical level (Fig. 3B).

### SEM analysis

**Figure 4:**
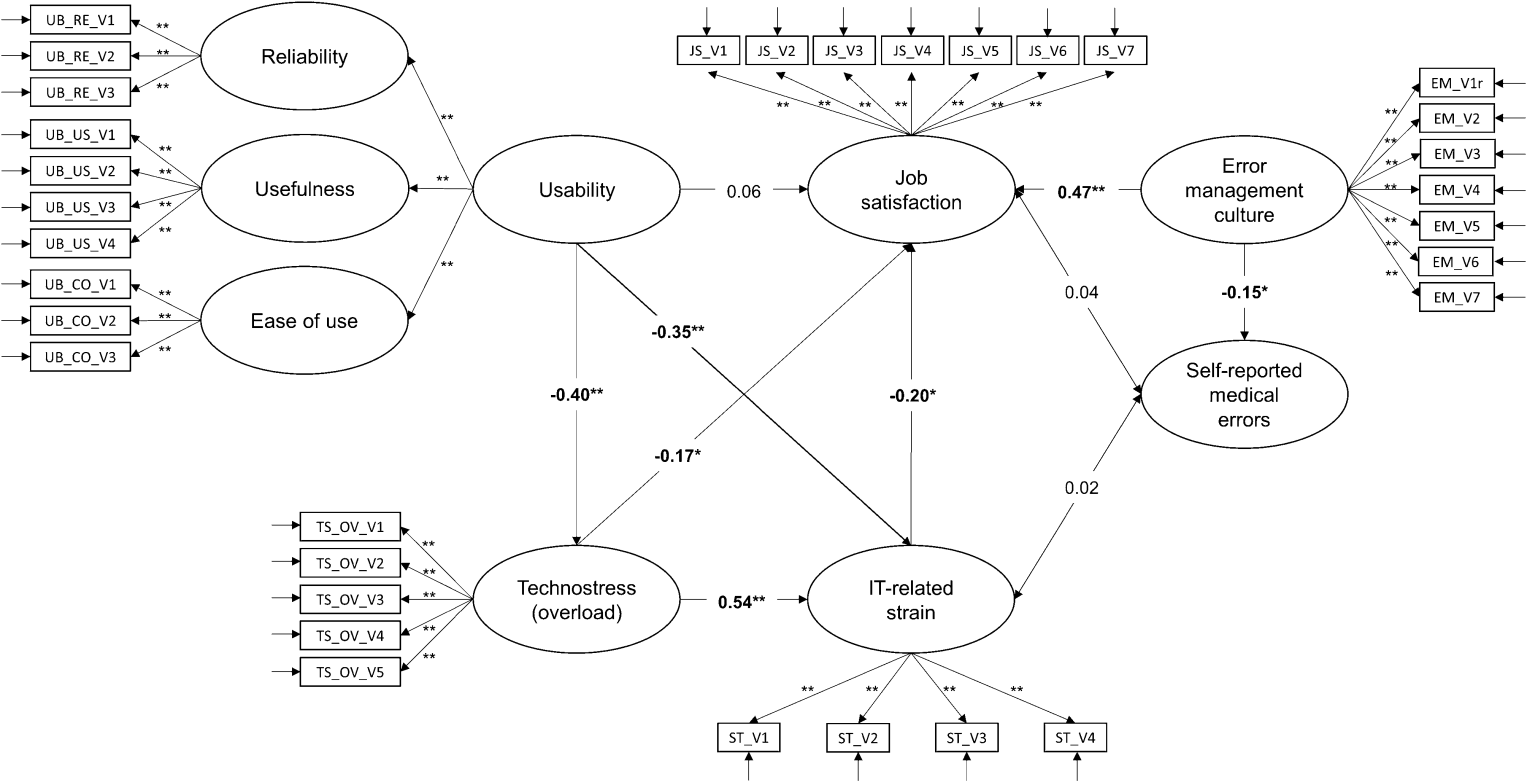
Structural equation model (measurement and structural model), *N* = 445, *p < .050, **p < .001.

Self-reported frequency of making a medical error was measured on an ordinal scale; therefore, lavaan automatically used the WLSMV estimator. The measurement model of the initially proposed model showed that items from the technology self-efficacy and techno-uncertainty scales had standardized factor loadings below 0.40. Additionally, techno-uncertainty did not load well on the overall technostress scale (0.26). Therefore, a second SEM model was calculated without technology self-efficacy as a moderator between technostress and strain (see a separate analysis below) and without the techno-uncertainty technostress subscale. With the new measurement model, all indicators (i.e., items) had significant positive factor loadings, with standardized coefficients ranging from 0.49 to 0.94, *p* < 0.001. The model fit the data very well: *χ*^*2*^ = 760.74, df = 514, *χ*^*2*^/df = 1.48, *p* < 0.001, RMSEA = 0.03 with 90% CI [0.28, 0.38], SRMR = 0.06, CFI = 0.98, and TLI = 0.98. Figure 4 shows the final model with standardized parameter estimates.

Perceived usability was negatively associated with experienced techno-overload (*p* < 0.001, 95% CI [− 0.52, − 0.20]), confirming the proposed hypothesis, and accounted for 16% of its variance. In accordance with our assumptions, usability correlated negatively with healthcare workers’ perceived strain (*p* < 0.001, 95% CI [− 0.68, − 0.35]), and techno-overload showed a positive association with strain (*p* < 0.001, 95% CI [0.63, 1.17]). Together, the usability and techno-overload variables explained 57% of the variance in reported IT-related strain. Against our hypothesis, usability did not significantly correlate with respondents’ job satisfaction (*p* = 0.346, 95% CI [− 0.08, 0.22]). However, in accordance with our proposed assumptions, participants’ reported job satisfaction was negatively associated with both techno-overload (*p* = 0.023, 95% CI [− 0.38, − 0.03]) and IT-related strain (*p* = 0.014, 95% CI [− 0.26, − 0.03]) and positively linked to error management culture (*p* < 0.001, 95% CI [0.37, 0.67]). Together, the four variables of usability, techno-overload, strain, and error management culture accounted for 41% of the job satisfaction’s variance. Neither perceived job satisfaction (*p* = 0.573, 95% CI [− 0.09, 0.15]) nor work-related strain (*p* = 0.745, 95% CI [− 0.07, 0.10]) emerged as significant predictors for participants’ self-reported frequency of making medical errors. However, in accordance with our hypothesis, a constructive error management culture was associated with fewer self-reported medical errors (*p* = 0.007, 95% CI [− 0.28, − 0.04]). Only 2% of the variance in participants’ self-reported frequency of making medical errors could be explained by the model. Job satisfaction correlated positively with a constructive error management culture (*p* < 0.001, 95% CI [0.37, 0.67]), confirming our assumption.

### Moderation analysis

We expected that participants’ levels of technology self-efficacy would moderate the relationship between perceived technostress and IT-related strain. The moderation analysis was performed by fitting a multiple regression with strain as the dependent variable and technostress (overload), technology self-efficacy, and the interaction term (technostress × technology self-efficacy) as predictors. We found a statistically significant albeit very small moderation effect *b* = − 0.07, *t*(441) = − 2.10, *p* = 0.030, *r* = − 0.10, 95% CI [− 0.19, 0.01]. This means that respondents with higher *technology self-efficacy* report slightly lower levels of *strain* and vice versa, which confirms the proposed hypothesis.

## Discussion

The current study investigated the associations between perceived HIT characteristics (usability and techno-stressors) and important organizational variables among German healthcare workers. The results extend the understanding of how the discerned usability of widely used HITs relates to perceived technostress, IT-related strain, job satisfaction, and self-reported medical errors. To the best of our knowledge, this is the first study to show the complex interactions between these variables using a SEM approach.

We found that hospital information systems, image archives, and electronic health records were the most widely used HITs. Good perceived usability (i.e., reliability, usefulness, and ease of use) of these HITs was associated with lower levels of techno-overload and IT-related strain. However, good usability of these HITs did not significantly correlate with job satisfaction. Participants who reported experiencing techno-overload (i.e., feeling that the HITs were forcing them to work faster/longer) were more likely to feel strained and slightly less satisfied with their job. The relationship between techno-overload and IT-related strain was somewhat moderated by a person’s level of technology self-efficacy. Facing IT-related strain was also directly linked with lower job satisfaction. Against our expectations, healthcare workers’ job satisfaction and perceived strain were not related to their self-reported frequency of making medical errors. However, perceiving the workplace’s error management culture as encouraging to workers to discuss the reasons and consequences of errors and motivating workers to learn from errors was associated with a lower frequency of medical errors. Rating the error management culture at work as constructive was very strongly associated with higher job satisfaction.

We want to highlight that the model accounted for 41% of the respondents’ job satisfaction variance. Job satisfaction is one of the most widely researched organizational psychology topics, consistently related to subjective wellbeing and many work-related behaviors such as turnover decisions, prosociality, organizational citizenship, counterproductive work behaviors, and job performance^19^. Our findings correspond to other studies that found that technostress and IT-related strain correlate negatively with job satisfaction^8,16–18,20–22^. Based on the literature, the direction of effects should be that exposure to techno-stressors leads to IT-related strain, which negatively impacts job satisfaction^11^. Again, in accordance with previous research, we found that good HIT usability is associated with lower levels of perceived technostress and IT-related strain and vice versa^10,20,26,27^. Therefore, HIT designers should focus on making their products reliable, useful for the end user, and easy to operate. Before and during the implementation of new HITs, their usability and potential for causing technostress should be assessed. For instance, in one study, a dashboard providing important data for diabetes care was developed with a user-centered design process, in which the end users were involved in initial focus groups, iterative feedback loops, and evaluation actives^59^. The usability evaluation showed that the new dashboard reduced the number of mouse clicks and the time to find all necessary data and increased the accuracy of acquiring data. To ensure that HIT-developers have an incentive to go through rigors pre-testing, policymakers and administrative bodies should set and enforce high usability standards for HITs during the approval process.

Workplace error management culture showed the strongest association with job satisfaction. The error management culture scale assessed whether healthcare workers felt that errors are being discussed and communicated openly and causes for errors are identified and resolved. These findings are in line with previous research showing that an effective error management culture is positively related to job satisfaction^47^. It has been argued that an effective error management culture can help employees control negative emotions in response to errors and increase motivation^46^, both of which can contribute to job satisfaction. Therefore, our findings highlight that healthcare facilities should invest in establishing a constructive error management culture in which staff are not afraid of being punished for causing and/or reporting errors.

Having an effective error management culture was not only associated with higher job satisfaction in our data; it was also a significant correlate with self-reported medical errors. This result was expected, considering previous research indicating that effective open error communication allows employees to learn from other people’s mistakes, preventing the same error from happening again in the future^45,46^. Other industries’ experience shows that an effective error management culture results in lower numbers of incidents. For instance, the aviation industry has a long history of gathering data and implementing effective processes such as checklists for error management, which could also be implemented in medicine and healthcare^60,61^. Scholars of error management have argued that healthcare can learn from the aviation industry’s strategies for enhancing teamwork and safety to establish effective error management programs^61^. Therefore, policymakers and administrative bodies should also set and enforce high standards for the reporting, evaluation, and management of medical errors to facilitate an effective error management culture.

The distribution of the medical error items needs further discussion. The participants’ reported frequency of making medical errors themselves was skewed towards the right, suggesting that no or only very few errors occurred in the last 3 months. This is somewhat surprising, considering that it was made clear to the participants that even minor mistakes should be viewed as medical errors. For instance, knowing that compliance with hand hygiene guidelines, a preventive behavior that has to occur countless times a day, is below 70% among German physicians^62^, it is unlikely that participants made no error in the last 3 months. Considering that 89.0% of our participants reported having made an error only once a month or even less often, we think it is plausible that the respondents did not account for minor errors when answering the question. This assumption is informed by other research that found only between 8.9 and 14.7% of physicians reported to have made a major medical error in the last 3 months^33,34,54^.

This might help to explain why we did not find significant associations between self-reported medical errors and job satisfaction or IT-related strain. Low job satisfaction and high IT-related strain are associated with the feeling of fatigue, which has been shown to result in errors of omission due to a lack of attention or concentration problems^63^, but might not result in major errors. More research is needed to test whether job satisfaction and IT-related strain might be linked to medical errors after all. Additionally, future research should be conducted to measure the occurrence of medical errors more reliably. Here it should be noted that the reported frequency of observing medical errors made by colleagues is much less skewed. It is not entirely clear why most participants think that their colleagues err more often than themselves. There might be well-established cognitive biases at play, such as the “better than average” effect, which describes people’s tendency to view their abilities above average, especially compared to their peers^64^. Or it could be that respondents just summed up errors they witnessed in their immediate work environment by multiple colleagues without making a comparison to their own error rate as a single person. Independent of the cause of the discrepancy between errors made by themselves and others, better measurements for the occurrence of medical errors are needed to study their sources. Using automatically collected data from technologies such as decision support systems, which register (potential) errors made by the user such as wrong dosages, might be one possible approach.

Several limitations of the present study should be mentioned. First, all measured variables were self-reported by participants and not objectively observed. For most variables, using questionnaire items is the standard way of measuring them. However, we are concerned that participants might have underreported the frequency of their own medical errors, considering how much higher they rated their colleagues’ error frequency. Future research on medical errors should try to use observable instead of self-reported data. Second, while we used well-established scales to measure techno-uncertainty, techno-insecurity, and technology self-efficacy, there were some concerns about their psychometric qualities, which led us to exclude them from the SEM. More research is needed to develop and validate scales for a wide range of target groups and multiple languages to ensure their reliability and validity. Third, the survey response rate was low, which is a common issue with online surveys^65^. A low response rate can pose a risk for a non-response bias in the results, which occurs when the group of non-responders differs in a meaningful way from the group of responders. Having a non-response bias in the data might affect the generalizability of the study’s results to the entire population. Finally, the study design was cross-sectional, which means that we cannot confirm the causal directions of the reported effects, and in several cases, both directions are theoretically plausible. Longitudinal studies and carefully planned experiments are needed to verify the causal links.

In conclusion, the present study leads to a deeper understanding of the perception of HIT characteristics and their relationship with organizational variables in healthcare. Our findings indicate that ensuring high usability of HITs is crucial to alleviate techno-overload and IT-related strain, which are both negatively linked to healthcare workers’ job satisfaction. Additionally, the results suggest that an effective error management culture helps ensure job satisfaction and reduce medical errors. Considering the strongly skewed self-reported frequency of making medical errors, future research needs to establish more objective ways to measure the occurrence of medical errors. Being able to measure medical errors objectively is an important first step towards constructively dealing with them and the basis for an effective error management culture. The present study results should help guide the successful implementation of HITs to realize their full potential for improving the quality of healthcare.

## Supplementary Information

Supplementary Information.

## Data Availability

The study’s pre-registration, data, survey material, and R-script will be made available online upon publication: https://osf.io/ekj9p/.
