# Author Correction: The relationship between health IT characteristics and organizational variables among German healthcare workers

**DOI:** 10.1038/s41598-021-98823-x

**Published:** 2021-09-21

**Authors:** Susanne Gaube, Julia Cecil, Simon Wagner, Andreas Schicho

**Affiliations:** 1 Department of Infection Prevention and Infectious Diseases, University Hospital Regensburg, Regensburg, Germany; 2 LMU Center for Leadership and People Management, LMU Munich, Munich, Germany; 3 Department of Psychology, University of Regensburg, Regensburg, Germany; 4 Department of Radiology, University Hospital Regensburg, Regensburg, Germany

Correction to: *Scientific Reports* 10.1038/s41598-021-96851-1, published online 07 September 2021

The Funding section in the original version of this Article was incomplete.

“Open Access funding enabled and organized by Projekt DEAL.”

now reads:

“Open Access funding enabled and organized by Projekt DEAL. The VolkswagenStiftung provided additional Open Access funding for this publication (Grant Number: 98 525).”

The original Article has been corrected.

