# Supplementary material for: The relationship between health IT characteristics and organizational variables among German healthcare workers
Source: Sci Rep. 2021 Sep 7;11:17752. doi: 10.1038/s41598-021-96851-1 (PMC8423839; doi:10.1038/s41598-021-96851-1)
Supplement: Supplementary file 1 — Supplementary Information. [file 41598_2021_96851_MOESM1_ESM.pdf]

SUPPLEMENTARY INFORMATION

for

The relationship between health IT characteristics and organizational variables among German healthcare workers

Susanne Gaube[1,2,3*], Julia Cecil[2,3], Simon Wagner[3], and Andreas Schicho[4]

Author Note

[1]Department of Infection Prevention and Infectious Diseases, University Hospital Regensburg, Regensburg, Germany.

[2]LMU Center for Leadership and People Management, LMU Munich, Munich, Germany.

[3]Department of Psychology, University of Regensburg, Regensburg, Germany.

[4]Department of Radiology, University Hospital Regensburg, Regensburg, Germany.

*Correspondence to: susanne.gaube@ukr.de

The file contains:

Table S1: List of scales and items used in the survey

Figure S1: Most widely used health information technologies

Table S1

List of scales and items used in the survey

Scale<br>Items	*M* (*SD*)
**Technologies in use**	n.a.
Instruction: Which of the following health information technologies (HITs) do you regularly use in your daily work? Multiple selections are possible. Note: The names of the technologies vary with the providers, so only over-categories are given.	
Electronic health record	
Hospital information system	
Image archive	
Radiology information system	
Mobile digital medical technology	
Large digital devices	
Laboratory information system	
Others	
**Usability: Usefulness**	5.26 (1.46)
Use of HITs enables me to accomplish tasks more quickly.	
Use of HITs improves the quality of my work.	
Use of HITs makes it easier to do my job.	
Use of HITs enhances my effectiveness on the job.	
**Usability: Ease of use**	4.9 (1.24)
Learning to use HITs is easy for me.	
HITs are easy to use.	
It is easy to get the results that I desire from HITs.	
**Usability: Reliability**	4.22 (1.47)
The features provided by HITs are dependable.	
The capabilities provided by HITs are reliable.	
HITs behave in a highly consistent way.	
**Technostress: Techno-overload**	3.45 (1.47)
I am forced by this HIT to work much faster.	
I am forced by this HIT to do more work than I can handle.	
I am forced by this HIT to work with very tight time schedules.	
I am forced to change my work habits to adapt to new HITs.	
I have a higher workload because of increased technology complexity.	

Scale<br>Items	*M* (*SD*)
**Technostress: Techno-uncertainty**	3.97 (1.20)
There are always new developments in the technologies we use in our organization.	
There are constant changes in computer software in our organization.	
There are constant changes in computer hardware in our organization.	
There are frequent upgrades in computer networks in our organization.	
**Technostress: Techno-insecurity**	1.73 (0.85)
I have to constantly update my skills to avoid being replaced.	
I am threatened by coworkers with newer technology skills.	
I do not share my knowledge with my coworkers for fear of being replaced.	
I feel there is less sharing of knowledge among coworkers for fear of being replaced.	
**Technology self-efficacy**	5.07 (1.07)
When I have to learn a new task that is high-tech, my first reaction is that I'm sure I can do it.	
In terms of my ability to learn new tasks that are high-tech, I would describe myself as one of the best in my workgroup.	
In the past, I have had a great amount of experience (either on or off the job) working on high-tech tasks.	
I am extremely confident that I can learn to use HITs on my job.	
HITs will allow me to perform my job better and more efficiently.	
**Strain**	2.57 (1.48)
I feel drained from activities that require me to use HITs.	
I feel tired from my HITs activities.	
Working all day with HITs is a strain for me.	
I feel burned out from my HITs activities.	
**Job satisfaction**	4.84 (1.18)
Instruction: Regarding your work in general. How pleased are you with….	
... your work prospects?	
… the people you work with?	
… the physical working conditions?	
… the way your department is run?	
… the way your abilities are used?	
… your usual take-home pay?	
... your job as a whole, everything taken into consideration?	

**Scale**<br>Items	*M* (*SD*)
**Error management culture**	4.58 (1.41)
Out of fear of reprimand, errors are covered up. (R)	
Errors are discussed openly with colleagues.	
Errors are discussed openly with superiors.	
Errors are communicated openly to patients and relatives.	
Errors are systematically recorded and documented.	
Possible causes for errors are discussed.	
Causes of errors are eliminated systematically if possible.	
**Common types of medical errors**	n.a.
Error in diagnosis or delay in diagnosis	
Failure to employ indicated tests	
Use of outmoded tests or therapy	
Failure to act on the results of monitoring or testing	
Technical error in the performance of an operation, procedure, or test	
Error in administering the treatment (including preparation for treatment or operation)	
Error in the dose of a drug or in the method of using a drug	
Avoidable delay in treatment or in responding to an abnormal test	
Inappropriate (not indicated) care	
Failure to provide indicated prophylactic treatment	
Inadequate monitoring or follow-up of treatment	
**Self-reported medical errors (self)**	2.19 (1.14)
How often have you made medical errors in the last 3 months?	
**Consequences of last medical error**	2.72 (2.39)
What was the outcome of your most recent error?	
**Self-reported medical errors (others)**	3.48 (1.58)
How often have you noticed medical errors made by your colleagues in the last three months?	

**Scale**<br>Items	*M* (*SD*)
**Perceived reasons for medical errors: Individual and organizational level**	n.a.
Instruction: On an individual and organizational level, what do you think are the top three causes of medical errors?	
Lack of knowledge, training, or experience	
Poor communication	
Incorrect risk assessment	
Inadequate self-reflection / questioning one's own decisions	
Distractions and interruptions	
Fatigue / exhaustion	
Revision / workload / time pressure	
Insufficient resources (e.g., staff)	
Incorrect or missing standards / protocols / guidelines	
Inadequate patient participation	
Interpersonal conflicts (e.g., with colleagues or superiors)	
Other	
**Perceived reasons for medical errors: technological level**	n.a.
Instruction: On a technological level, what do you think are the top three causes of medical errors?	
There is a lack of instruction and training on how to use the technologies	
I cannot understand how the technologies work	
I have to align my workflow too much with the requirements of technology	
Technologies take my control and limit my freedoms	
The technologies do not work with the common terms and standards	
Technologies don't adequately warn me of impending errors	
The technologies are constructed illogically	
The amount of setting options prevents me from doing my work quickly and adequately	
The user interface is not well designed	
The user interface shows me too much unnecessary information	
Questions regarding the operation of the technologies are not adequately answered	
The technologies don't work reliably	
Other	

Note. N = 445, HITs = health information technologies, n.a. = not applicable

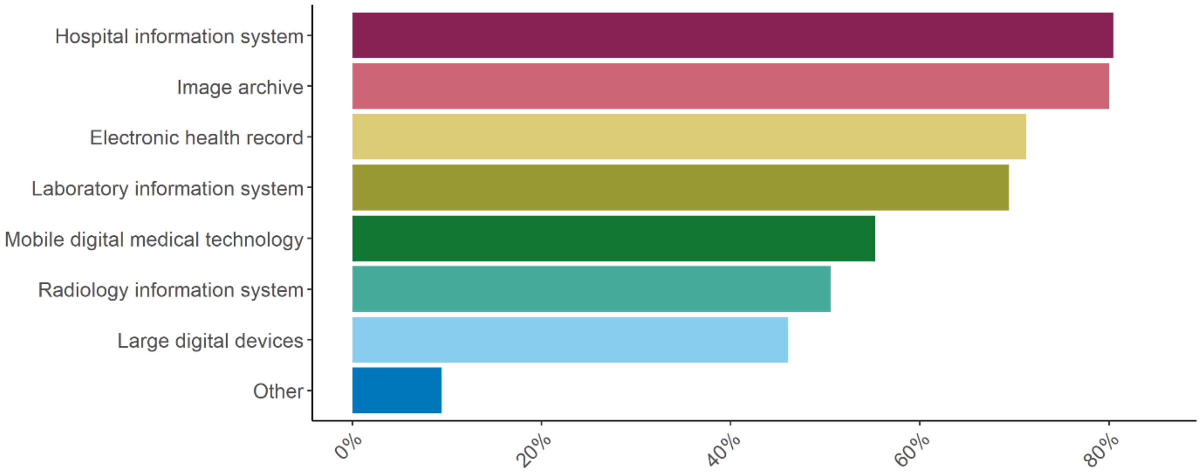

Figure S1. Most widely used health information technologies
